# Supplementary material for: Barriers and Facilitators of Fruit and Vegetable Consumption in Renal Transplant Recipients, Family Members and Healthcare Professionals—A Focus Group Study
Source: Nutrients. 2019 Oct 11;11(10):2427. doi: 10.3390/nu11102427 (PMC6835653; doi:10.3390/nu11102427)
Supplement: Supplementary file 1 [file nutrients-11-02427-s001.pdf]

Supplementary material

S1. Example of an interview guide of the focus groups with RTR.

1. Can you describe how your life has changed after renal transplantation?
2. What influences does the renal transplantation have on your eating and drinking habits?

   Probes:

   - Could you describe what you are allowed to eat? (K)
   - Does medication influence your eating and drinking habits?
   - And the dialysis time before transplantation?
   - How did you receive / obtain information about nutrition after transplantation? (K)
   - What kind of information sources did you use? (K)

3. What is the role of eating and drinking in your daily life?

   Probe:

   - How important is healthy eating after transplantation? (K)
   - Are you satisfied with your current dietary pattern? (G)

4. How would it be to eat more fruit and vegetables after transplantation? (G)

   Probe:

   - What are the potential benefits? (OE)
   - Do you think you're able to eat more fruit and vegetables? (SE)
   - If it is difficult to change, what can support you? (S)

5. What makes it difficult to eat fruit and vegetables? (B)
6. What makes it easier to eat fruit and vegetables? (F)

   Probes (question 5 and 6)

   - Could you give an example?
   - What is the role of your environment, e.g. your family? (S)
   - What is the role of financial resources?

The discussion guide is in line with the core concepts of the social cognitive theory: (K), knowledge; (G), goals; (OE), outcome expectancies; (SE), self-efficacy; (S), social support; (B), barriers; (F), facilitators
